# Identification of a novel K311 ubiquitination site critical for androgen receptor transcriptional activity

**DOI:** 10.1093/nar/gkw1162

**Published:** 2016-11-28

**Authors:** Urszula L. McClurg, David M.W. Cork, Steven Darby, Claudia A. Ryan-Munden, Sirintra Nakjang, Leticia Mendes Côrtes, Achim Treumann, Luke Gaughan, Craig N. Robson

**Affiliations:** 1 Solid Tumour Target Discovery Laboratory, Newcastle Cancer Centre, Northern Institute for Cancer Research, Medical School, Newcastle University, Newcastle upon Tyne NE2 4HH, UK; 2 Bioinformatics Support Unit, Faculty of Medical Sciences, Newcastle University, Newcastle upon Tyne, UK; 3 Newcastle University Protein and Proteome Analysis, Devonshire Building, Devonshire Terrace, Newcastle upon Tyne NE1 7RU, UK

## Abstract

The androgen receptor (AR) is the main driver of prostate cancer (PC) development and progression, and the primary therapeutic target in PC. To date, two functional ubiquitination sites have been identified on AR, both located in its C-terminal ligand binding domain (LBD). Recent reports highlight the emergence of AR splice variants lacking the LBD that can arise during disease progression and contribute to castrate resistance. Here, we report a novel N-terminal ubiquitination site at lysine 311. Ubiquitination of this site plays a role in AR stability and is critical for its transcriptional activity. Inactivation of this site causes AR to accumulate on chromatin and inactivates its transcriptional function as a consequence of inability to bind to p300. Additionally, mutation at lysine 311 affects cellular transcriptome altering the expression of genes involved in chromatin organization, signaling, adhesion, motility, development and metabolism. Even though this site is present in clinically relevant AR-variants it can only be ubiquitinated in cells when AR retains LBD suggesting a role for AR C-terminus in E2/E3 substrate recognition. We report that as a consequence AR variants lacking the LBD cannot be ubiquitinated in the cellular environment and their protein turnover must be regulated via an alternate pathway.

## INTRODUCTION

Prostate cancer (PC) is the most common male malignancy in developed countries accounting for 15% of newly diagnosed male cancer cases in 2012 (World Cancer Research Fund International). Globally, in 2012 an estimated 307 000 men died from PC with deaths resulting from advanced, metastatic disease. PC is driven by the androgen receptor (AR) and tumours will initially respond to androgen deprivation therapy (ADT). However, the main clinical challenge for PC results from the inevitable recurrence of resistant tumours, termed castrate resistant prostate cancer (CRPC) for which there are no curative treatments and prognosis is extremely poor. Recently developed second generation anti-androgens are characterized by a response rate of only 50% highlighting the need for new targeted therapies (1). The AR remains a crucial driver in CRPC, which results in common therapy-induced amplification and over-expression of full-length (FL) and (alternatively spliced/truncated) AR variants (AR-V), AR mutations and increased expression of AR-regulated genes including PSA. Interestingly, an increase in AR mRNA/protein level is the only consistently observed molecular change in CRPC (2). The AR is a ligand-inducible transcription factor that regulates genes involved in prostate growth and transformation (3). Following androgen binding, cytoplasmic AR is phosphorylated promoting conformational change resulting in dimerization, chaperone protein dissociation and nuclear translocation. Nuclear AR binds androgen-responsive genes and activates cellular transcription. We and others have demonstrated numerous post-translational modifications of AR that regulate its transcriptional output (4). The intricate balance between positive and negative AR co-regulators is also modulated by components of the ubiquitin–proteasome system.

Ubiquitination is a reversible covalent process that results in an attachment of a ubiquitin molecule to specific substrates that regulates multiple cellular processes, including protein degradation (5). Ubiquitin attachment is mediated by sequential activating (E1), conjugating (E2) and ligase (E3) enzymes. Ubiquitin contains seven acceptor lysine residues (K) which enable poly-ubiquitin chain formation (6) and one intrinsic Met1 residue that enables linear ubiquitin chain formation (7). The position of the lysine residue within ubiquitin that is used for ubiquitin chain branching dictates the fate of the substrate. For example, K48 poly-ubiquitin chains primarily target proteins for proteasomal degradation whilst K63 chains primarily mediate cellular processes, including transcription factor activity, protein–protein interaction and DNA repair (8). Ubiquitin can be removed from target proteins by deubiquitinating (DUB) enzymes (9). Dysregulation of the ubiquitin–proteasome pathway occurs in PC pathogenesis and multiple AR partners are components of the ubiquitin–proteasome degradation system. Ligand binding promotes AR ubiquitination and destruction suggesting that AR turnover and transcriptional activity are coupled (10).

Currently, two AR ubiquitination sites have been identified at K845 and K847, both in the C-terminal ligand binding domain (LBD) of AR (11). Several E3 ligases have been implicated in AR biology with AR ubiquitination commonly controlling its protein stability. MDM2 was the first E3 found to ubiquitinate AR with AR phosphorylation being a prerequisite for this process which consequently leads to AR protein degradation (12). In the absence of this phosphorylation AR is ubiquitinated by CHIP instead (13). SPOP was also implicated in AR destruction following recognition of an S/T-containing degron and commonly identified PC-linked SPOP mutants fail to bind AR to promote its destruction (14,15). Similarly, most constitutively active AR variants (AR-V) lacking the ligand binding domain which are linked to CRPC are not degraded by SPOP. Alternatively, AR ubiquitination can regulate receptor activity, RNF6 poly-ubiquitinates AR with K6/K27 linked chains enhancing AR transcriptional activity through co-activator recruitment. Consequently RNF6 is overexpressed in CRPC and promotes growth under androgen-depleted conditions (11). These AR modifications can be reversed by DUB enzymes, including USP26 (16) and USP12 (17,18).

In this manuscript, we report the discovery of a novel N-terminal domain (NTD) AR ubiquitination site at K311 which regulates both AR protein stability and is also critical for AR transcriptional activity and binding to p300 in the presence of androgen. Mutation of this site causes retention of AR on chromatin and abrogates its activity toward target genes. When this ubiquitination site is lost transcription of over a thousand genes is altered with global changes to gene expression in chromatin organization, cellular adhesion and motility, signal transduction, development and cellular metabolism pathways. Additionally, we report that AR-Vs are not ubiquitinated and hence their activity is neither regulated through this site nor by the MDM2 E3 ligase.

## MATERIALS AND METHODS

### Antibodies and plasmids

anti-AR (Santa Cruz Biotechnology; N20 and C19 clones), anti-AR-v7 (Precision Antibodies), anti-H2A.Z (19), anti-MDM2 (Santa Cruz N20 and SMP14), anti-HA (Santa Cruz Biotechnology; Y11 clone), anti-FLAG (Sigma), anti-p53 (Dako), anti-α-tubulin (Sigma), and anti-ubiquitin (Santa Cruz Biotechnology) were included in this project. Plasmids used were pPSA-Luc, pARE3-Luc, pCMV-β-gal, pHA-ubiquitin, pMYC-MDM2, pFLAG-His-AR (20) and pFLAG-His-AR-K311R and pFLAG-V7-AR mutants generated by *in vitro* mutagenesis (QuikChange; Stratagene). Lentiviral AR careers were prepared as previously described (21).

### Cell culture, transfections, and reporter assays

LNCaP, HEK293T, CWR22Rv1 and COS-7 cells (all purchased from the ATCC) were cultured in RPMI 1640 medium with 2 mM l-glutamine (Invitrogen) supplemented with 10% (v/v) foetal calf serum (FCS) at 37°C in 5% CO_2_. Transfections were performed using TransIT-LT1 reagent (MirusBio) following the manufacturer's instructions.

For luciferase assays, cells were transfected with 50 ng of pARE3-luc, 50 ng of pCMV-β-gal and 10 ng of pFLAG-His-AR and pMYC-MDM2 as required. All reactions were balanced with pCMV empty vector. Cells were cultured in steroid-depleted media (SDM) for 48 h followed by supplementation with 10 nM dihydrotestosterone (DHT) for an additional 24 h. Cells were lysed and incubated in 1× Promega luciferase assay reagents according to the manufacturer's instruction, and luciferase counts were established and normalized to β-galactosidase activity. Results were normalized to AR expression alone in steroid depleted conditions.

### *In vitro* ubiquitination assay and mass spectrometry analysis

*In vitro* ubiquitination assays were performed on purified N-terminal AR protein (aa 1–518) using a Ubiquitin Conjugation Reaction Buffer Kit (SK10) and incorporating the following enzymes: UBE1 (E-305), UBCH5 (E2–616) and MDM2 (E3–204) (all purchased from Boston Biochem) according to manufacturer's instructions. Monoubiquitination assays were performed using a modified ubiquitin lacking the seven lysine ubiquitin acceptor sites, Ub-K0 (UM-NOK) and polyubiquitination assays performed using wild type ubiquitin (U-100H). The E3 Ligases CHIP, E6AP and hPIRH2 were generated via GST purification and *in vitro* ubiquitination reactions repeated, reactions utilizing purified E3 ligases were conducted in the presence of Ubiquitin aldehyde (Cat U-201) to inhibit any potential deubiquitinase contamination from the E3 purification stage. All reactions were activated by ATP and quenched with E1 stop buffer.

Ubiquitination of N-terminal AR was confirmed via western blot using anti-AR and anti-ubiquitin antibodies (as outlined). Samples submitted for proteomic analysis were resolved via PAGE and stained using a silver stain II kit (Sigma) according to manufacturer's instructions or with coomassie stain. Bands representing monoubiquitinated AR were excised and sent for analysis at Newcastle University Protein and Proteome Analysis (NUPPA) facility.

### Proteomic analysis

#### In gel tryptic digest

The excised gel piece was put into a small volume of NH_4_HCO_3_ pH 7.8 and mashed to small pieces. The gel pieces were washed with 60% acetonitrile and washed with NH_4_HCO_3_ pH 7.8 prior to reduction in 50 μl of 10 mM DTT in 100 mM NH_4_HCO_3_ for 1 h at 56°C. Subsequently, cysteines were alkylated by adding 50 mM iodoacetamide in 100 mM NH_4_HCO_3_ and incubating in the dark at room temperature for 30 min. Gel pieces were repeatedly washed with 100 mM NH_4_HCO_3_ and 50% acetonitrile, and dehydrated using 100% acetonitrile. Proteins were in gel digested with 8 ng of trypsin (Promega) in 50 mM NH_4_HCO_3_, 1 mM CaCl_2_ and incubated overnight, shaking in a thermomixer at 37°C. The digest was stopped through addition of 5% of TFA. The gel pieces were washed twice with 60% acetonitrile containing 2% TFA and the washes were pooled with the aqueous supernatant after the digest. The volume of the digests was reduced to 10 μl in a speedvac and 10% was analysed by LCMSMS.

#### HPLC conditions

Peptides were concentrated on a Pepmap C-18 trap column (300 μm ID × 5 mm) and separated on a Pepmap C18 reversed phase column (Dionex, UK) (3 μm particles, 75 μm ID × 250 mm) using a linear gradient over 42 min from 96% A (0.05% formic acid), 4% B (0.05% formic acid, 80% acetonitrile) to 35% A, 65% B and a flow rate of 300 nl/min.

#### Mass spectrometry (MS) conditions

The spray voltage was 1.6 kV and the temperature of the heated capillary was set to 200°C. Survey scans were acquired with enabled lockmass from 400–1600 Da at a resolution of 30 000 at *m/z* 400 after accumulation of 5 × 10^5^ ions. The 10 most intense ions of the survey scan were sequenced concurrently to full scan acquisition in the orbitrap by collision-induced dissociation (CID, normalized collision energy 35%) in the LTQ. Maximum filling times were 50 ms for the full scans and 100 ms for the MSMS scans. Precursor ion charge state screening was enabled and unassigned charge states and singly charged ions were rejected. Dynamic exclusion was enabled for 180 s with a maximum dynamic exclusion list of 50 entries and a relative mass window from −0.5 Da to + 1 Da.

#### Immunoprecipitation (IP)

Cells were seeded at 10^6^ cells/90-mm dish, transfected 24 h later with 1 μg of each plasmid as indicated, incubated for 48 h, and lysed directly into lysis buffer (50 mM Tris, pH 7.5, 150 mM NaCl, 0.2 mM Na_3_VO_4_, 1% Nonidet P-40, 1mM PMSF, 1 mM DTT and 1× protease inhibitors (Roche Applied Science)). Lysates were incubated with 1 μg of antibodies as indicated for 16 h at 4°C, and antibodies were pulled down using protein G-Sepharose beads. For denaturing IPs, cells were subjected to 20 μM MG132 proteasomal inhibitor treatment for the final 16 h followed by collection into lysis buffer supplemented with 2% SDS and subsequently denatured at 100°C for 10 min (17). Immunoprecipitants were analyzed using western blotting.

For chromatin isolation, cells were trypsinized and resuspended in 1ml of Buffer A (10 mM HEPES pH 7.9, 10 mM KCl, 1.5 mM MgCl_2_, 0.34 M sucrose, 10% glycerol, 1 mM DTT and 1 × protease inhibitors cocktail) and incubated on ice for 10 min. Nuclei were collected by centrifugation at 1200 g for 5 min at 4°C. Supernatant was used as a cytosolic fraction and cleaned by centrifugation at 200 g for 5 min at 4°C. Nuclei were washed in 1 ml of buffer A without glycerol and resuspended in 1ml of buffer B (3 mM EDTA, 0.2 mM EGTA, 1 mM DTT and 1 × protease inhibitor cocktail) and incubated on ice for 30 min. Finally, sample was centrifuged at 1500 g for 5 min at 4°C and the chromatin pellet was washed in buffer B seven times followed by denaturing immunoprecipitation.

#### siRNA gene silencing and gene expression analysis

LNCaP cells were reverse transfected with siRNA using RNAiMax (Invitrogen) according to the manufacturer's instructions and incubated in culture medium for 96 h prior to cell lysis and analysis by western blotting as described previously (17) or qPCR. siRNA sequences were as follows SCR UUCUCCGAACGUGUCACGU[dT][dT]; full length AR CCAUCUUUCUGAAUGUCCU[dTdT]; AR UTR GCCAGCCACACAAACGUUU[dT][dT]. For RNA sequencing, RNA was extracted using the QIAGEN RNeasy Plus Mini Kit and all samples were sequenced in triplicate using Illumina's total stranded RNA prep kit with ribozero gold for library preparation with 100 bp paired end reads on the Illumina HiSeq 2500 platform, performed by AROS. Reads were mapped to the reference human genome hg38 using STAR (22). Per gene raw read counts for each sample were obtained using HTseq (23). Gene-level differential expression analysis was performed using DEseq2 (24). Differentially expressed genes from each comparison were tested for functionally enriched pathways and gene ontology terms using GOseq (25) and Enrichment Map (26).

#### qPCR

For qPCR RNA was extracted using Trizol (Invitrogen) according to manufacturer's instruction, and quantified using Nanodrop cDNA synthesis using the oligo dT primers and data analysis was performed as described previously (18). Briefly, 2 μg of RNA was reverse transcribed with 1ul of MMLV reverse transcriptase, 2 μl of 25nM Oligo-dT and 4 μl of 4mM dNTP (Promega). All qPCR was performed using the relative quantification method on three independent biological experiments and each sample was loaded in triplicate. qRT-PCR was conducted using SYBR^®^Green on 384-well optical reaction plates with the ABI 7900HT real-time PCR system. Results were normalized to HPRT1 expression. Primers were *KLK2* F: 5΄-AGCATCGAACCAGAGGAGTTCT-3΄, R: 5΄-TGGAGGCTCACACACTGAAGA-3΄; *HPRT1* F: 5΄-GAACGTCTTGCTCGAGAGATGTG-3΄, R: 5΄-CCAGCAGGTCAGCAAAGAATTT-3΄; *PSA* F: 5΄-ACTGCATCAGGAACAAAAGCGT-3΄, R: 5΄-TGTGGGAAGCTGTGGCTGAC-3΄; *NDRG1* F: 5΄-ACAACCCCCTCTTCAACTACG-3΄, R: 5΄-GCCAATAATGCTTTTCAGCCCA-3΄.

#### Chromatin immunoprecipitation (ChIP)

ChIPs were performed as described previously (27). LNCaP cells were transfected in steroid-depleted medium for 72 h followed by DHT treatment for 120 min. Data are presented as percentage input using the following formula: % input = 100 × AE (amplification efficiency)*(CT adjusted input sample – CT immunoprecipitated sample). CT refers to cycle threshold. ‘Walking ChiP’ was performed using primers representing different regions of the human *PSA* gene enhancer forward (F) 5΄-GGGGTTTGTGCCACTGGTGAG-3΄; reverse (R) 5΄-GGGAGGCAATTCTCCATGGTT-3΄; enhancer-promoter F 5΄-TAGAAGACGTGGAAGTAGCTG-3΄; R 5΄-AACCTCATGGATCCGGTGTCC-3΄; promoter F 5΄-TCTAGTTTCTGGTCTCAGAG-3΄; R 5΄-TTGCTGTTCTGCAATTACTAG-3΄; intron 1 F 5΄-CCAAGGACCTCTCTCAATGC-3΄; R 5΄-AGGGAATGAGGAGTTCTCAG-3΄; exon 3 F 5΄-CACACCCGCTCTACGATATGAG-3΄; R 5΄-GAGCTCGGCAGGCTCTGACAG-3΄; 3΄ UTR (untranslated region) F 5΄-TACTGGCCATGCCTGGAGAC-3΄; R 5΄-TGGCTCACAGCCTTCTCTAG-3 as previously described by Lee *et al.* (28); *KLK2* F: 5΄-ACCCCTGTTGCTGTTCATCCTG-3΄ R: 5΄-CCGCCCTTGCCCTGTTGG-3΄; *TMPRSS2* F: 5΄-TGGTCCTGGATGATAAAAAAAGTTT-3΄, R: 5΄-GACATACGCCCCACAACAGA-3΄.

#### Immunofluorescence

For Immunofluorescence, cells were cultured in steroid depleted media (SDM) for 72 h followed by 10 nM DHT treatment for 2 h. Cell were fixed for 20 min in 4% formaldehyde, permeabilized for 20 min in 0.1% Triton X-100 in PBS followed by blocking in 1% BSA, staining and imaging using Zeiss LSM 700 confocal microscope.

## RESULTS

### *In vitro* identification of a novel K311 ubiquitination site

**Figure 1.**
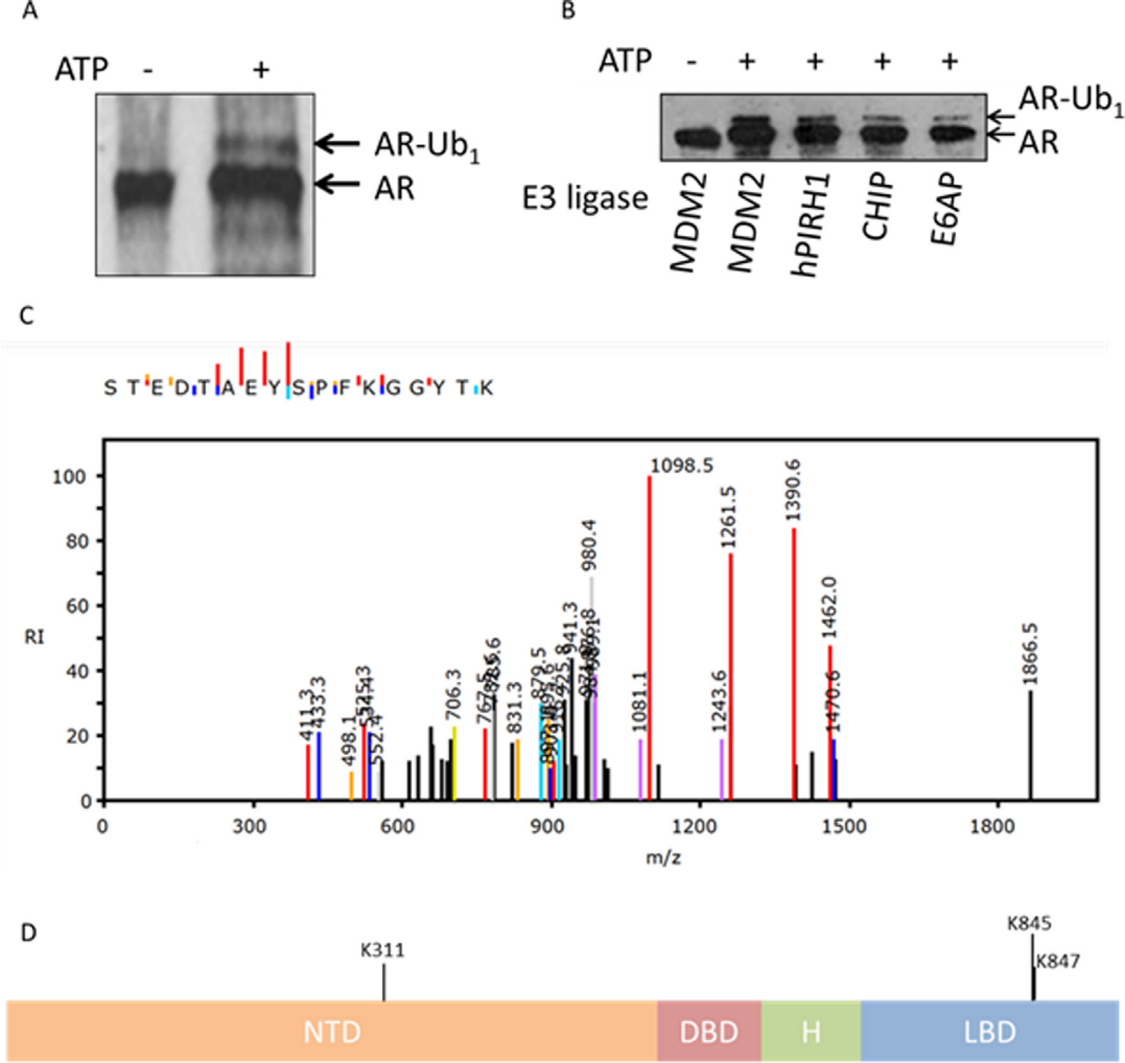
*In vitro* identification of K311 as a site of AR ubiquitination. (**A**) *In vitro* monoubiquitination of AR by MDM2 upon addition of ATP. Monoubiquitinated AR (AR-Ub1) was excised from the colloidal-coomassie stained gel for MSMS analysis. (**B**) Monoubiquitination of AR-NTD protein (AR-Ub1) can be detected by Western blotting following *in vitro* ubiquitination reactions in the presence of ATP, UBE1, UBCH5α and UbK0 using four different E3 ligases; MDM2, hPIRH2, CHIP and E6AP. (**C**) MSMS spectrum of the tryptic peptide, <302>STEDTAEYSPFK*GGYTK<318>, observed following in gel tryptic digest. Analysis of the spectrum locates unequivocally a diglycine modification to K311. (**D**) AR domain structure highlighting the novel site of AR ubiquitination, K311, within the N-terminal domain (NTD). Previously identified RNF6 target lysines within the ligand binding domain (LBD) are also indicated, K845 and K847. DBD—DNA binding domain, H—hinge.

Two AR ubiquitination sites (K845/K847) were recently identified in the C-terminus, both targeted by RNF6 (11). Previously we demonstrated AR ubiquitination by MDM2 in cell line experiments, but the sites of this AR modification were unknown (10). Given that several E3 ligases interact with AR, it is likely that receptor ubiquitination is catalysed by multiple enzymes (4). To test this, we established *in vitro* ubiquitination assays, incorporating purified N-terminal AR fragment (aa1-538), E1 (UBE1), E2 (UBCH5α) and various E3 ligase enzymes potentially linked to AR function to examine specificity of AR ubiquitination. By incorporating a modified ubiquitin lacking all seven lysine ubiquitin acceptor sites (UbK7R), we demonstrated AR N-terminal mono-ubiquitination by MDM2 (Figure 1A–B), PIRH2, CHIP and to a lesser extent E6-AP (Figure 1B). Mass spectrometry analysis of the MDM2 (and PIRH2)-mediated mono-ubiquitinated N-terminal AR species identified K311 as an ubiquitin acceptor (Figure 1C), highlighting the AR N-terminus as an E3 target (Figure 1D).

### AR K311 is not the sole MDM2-mediated ubiquitination site

**Figure 2.**
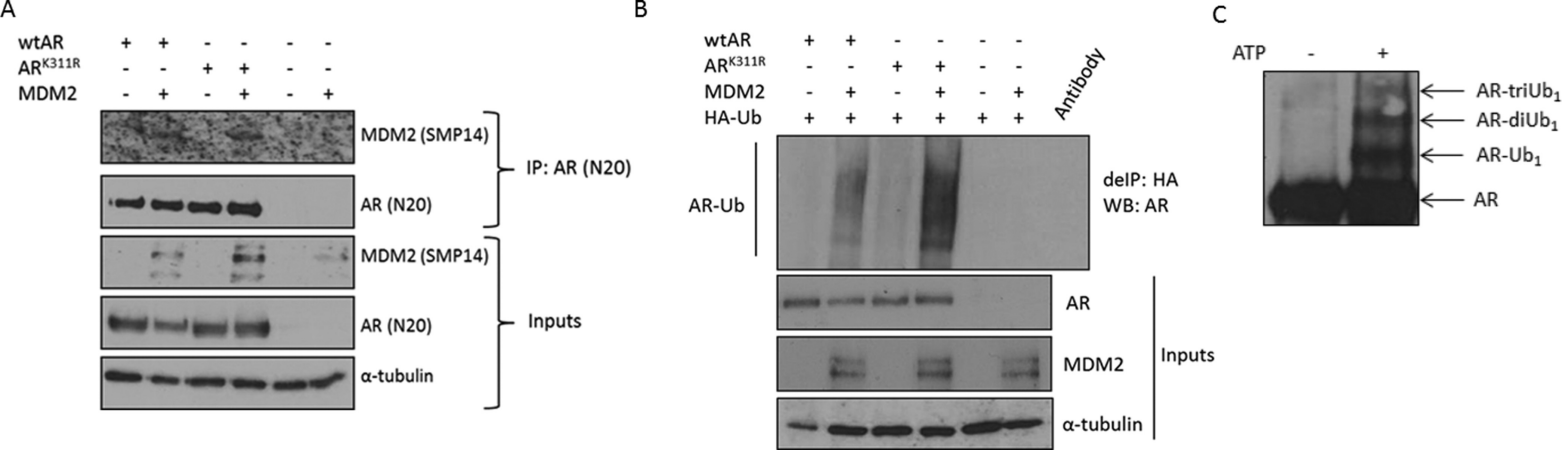
AR K311 is not the sole target of MDM2-mediated ubiquitination. (**A**) HEK293T cells were transfected as indicated and AR with interacting partners were immunoprecipitated followed by immunoblotting. (**B**) HEK293T cells were transfected as indicated followed by denaturing immunoprecipitation and immunoblotting. (**C**) Western blotting of AR DBD/H/LBD with annotated monoubiquitination (AR-Ub1) and di-monoubiquitination (AR-diUb1) by MDM2 following *in vitro* ubiquitination assay. Reactions contained UBE1, UBE2D3, and UbK0 in the absence or presence of ATP to activate the ubiquitination reaction.

MDM2 E3 ligase has been previously implicated in AR ubiquitination. To determine whether the K311 ubiquitination site is a target of MDM2 we mutated this site to arginine. Firstly, we assessed the ability of K311R AR to interact with MDM2 and we found that this interaction was similar between WT AR and mutant K311R AR (Figure 2A). Additionally, MDM2 was able to efficiently ubiquitinate AR lacking this site confirming that although K311 AR can be targeted by MDM2 it is not the sole target site of MDM2-mediated ubiquitination (Figure 2B). This is supported by additional *in vitro* ubiquitination experiments with MDM2 and a carboxy-terminal AR construct comprising both DNA binding domain (DBD) and ligand binding domain (LBD) which demonstrated a strong ubiquitination of this C-terminal construct by MDM2 (Figure 2C). We have not yet been able to verify the C-terminal site(s) of ubiquitination by MDM2 using MSMS.

### K311 ubiquitination plays a role in AR protein stability and is critical for its transcriptional activity

**Figure 3.**
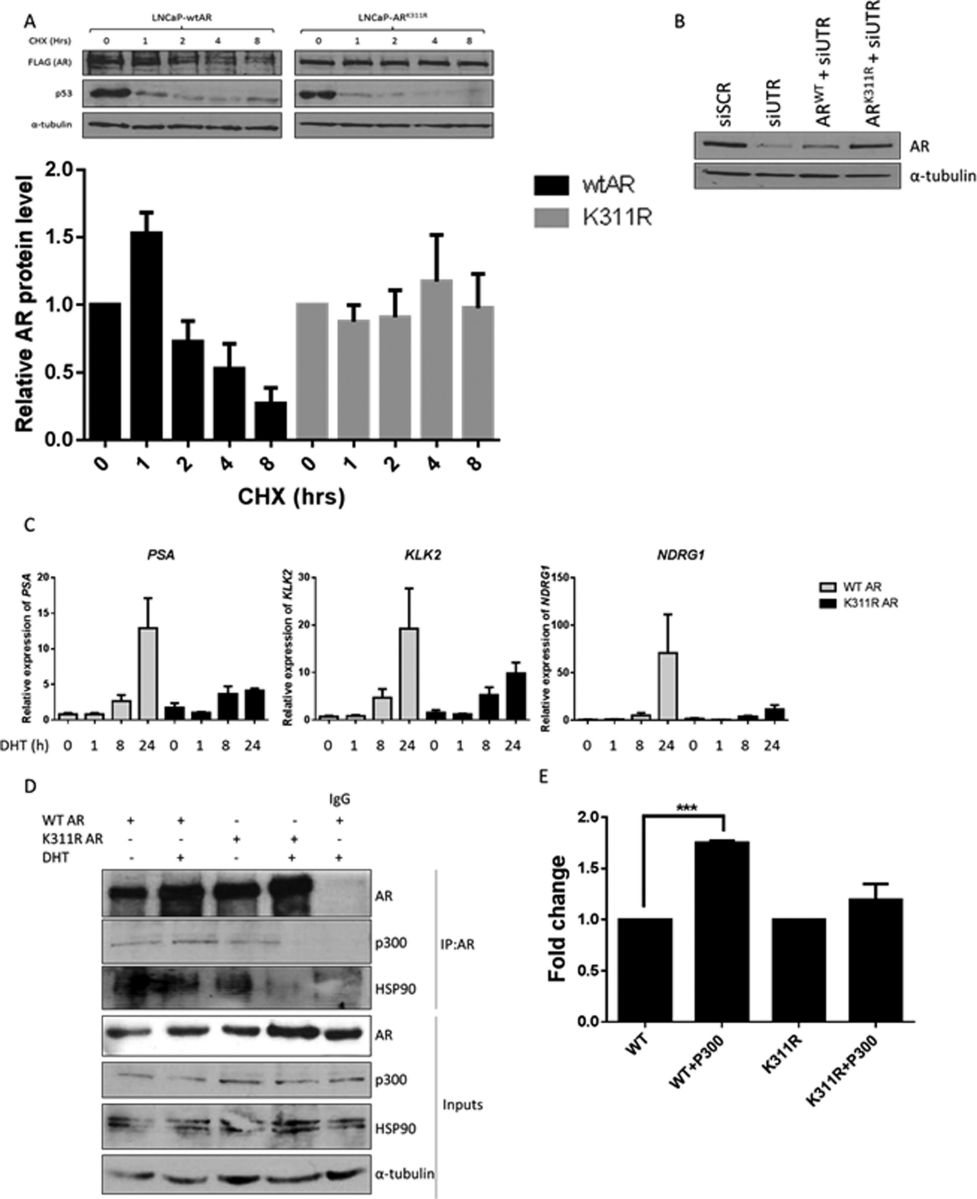
K311 is important for AR protein stability and plays a critical role for its transcriptional activity. (**A**) LNCaP cells stably expressing either FLAG-WT AR or FLAG-AR K311R were subjected to treatment with 10 μg/ml cycloheximide (CHX) as indicated, followed by Western blotting with an anti-FLAG antibody. Band intensity was quantified and graph represents three independent experiments ± SEM. (**B**) Silencing of endogenous AR using siRNA targeting the 3΄ UTR (siUTR) sequence absent in exogenous AR carrying vectors causes reduction in AR protein in non-transduced LNCaP cells compared to scrambled control siRNA (siScr). Cells overexpressing lentiviral WT AR or AR K311R show increased levels of exogenous AR compared to non-transduced LNCaP cells after silencing their endogenous AR with siUTR treatment. (**C**) qRT-PCR analysis of AR target gene expression in LNCaP cells stably overexpressing WT or K311R AR after silencing endogenous AR. Cells were cultured in steroid depleted media for 72 h followed by 24h treatment with 10 nM DHT. Data is a mean of three independent experiments normalized to HPRT1 ± SEM. (**D**) AR was immunoprecipitated from the LNCaP cells stably overexpressing WT or K311R AR cultured in the presence or absence of 10 nM DHT followed by immunoblotting. (**E**) HEK293T cells were transfected with WT or AR K311R alongside a luciferase reporter plasmid under the control of an androgen response sequence (ARE3) of the PSA promoter with and without p300 overexpression. Cells were cultured in steroid depleted media for 48 hours before 24 h treatment ± 10 nM DHT. Data represent a mean of three independent experiments ± SEM. Luciferase activity was normalized to each AR alone.

To determine whether the K311 ubiquitination site contributed toward AR protein turnover we assessed AR protein stability in the LNCaP cells stably overexpressing a lentiviral AR^WT^ or AR^K311R^ constructs by subjecting them to cycloheximide treatment. Relative to p53 which demonstrated the expected similar rapid turnover in both LNCaP derivative cell lines, the wildtype AR had an approximate half-life of 6 h whereas the AR K311R half-life was extended beyond 8 h (Figure 3A). This indicated that the K311 site is important for ubiquitination mediated AR protein turnover. To test the importance of the K311 ubiquitination site for AR transcriptional activity we created a lentiviral expression model of WT and K311R AR. We used siRNA targeting the endogenous 3΄ UTR sequence of AR (absent in both exogenously overexpressed WT and K311R AR constructs), to ensure endogenous AR silencing (Figure 3B, lanes 1 and 2). Subsequently we used LNCaP cells stably overexpressing the WT or K311R AR lentiviral vectors and silenced their endogenous AR using the siUTR sequence (Figure 3B, lanes 3 and 4). Using this model we compared androgen driven gene expression in LNCaP cells (lanes 3 and 4 of Figure 3B). We observed that even though, we had higher levels of K311R AR protein compared to WT AR (Figure 3B), due to increased protein stability (Figure 3A), this mutant failed to effectively induce transcription of AR target genes in the androgen dependent LNCaP cells indicating the critical importance of K311 for AR transcriptional activity (Figure 3C). To determine the reasons behind the lack of transcriptional activity of the K311R AR we assessed its ability to bind to AR co-factors. In the absence of androgen K311R had a very similar binding pattern to WT AR however, upon the addition of androgen, binding to both p300 and HSP90 was abrogated (Figure 3D). HSP90 is responsible for controlling AR stability in the cytoplasm (29) whereas p300 was previously shown to be required for both androgen dependent and independent AR activation. p300 is not only crucial for AR activation but is also required for AR polyubiquitination and degradation (30). Lack of K311R AR binding to p300 in the presence of androgen is the likely cause of increased K311R AR stability coupled with lack of transcriptional activity. This was further confirmed by a gene expression assay where transcriptional activity of WT AR was significantly enhanced by the addition of p300 while it had no effect on the activity of K311R AR (Figure 3E).

### AR protein lacking the K311 ubiquitination site is transcriptionally inactive due to its chromatin retention

**Figure 4.**
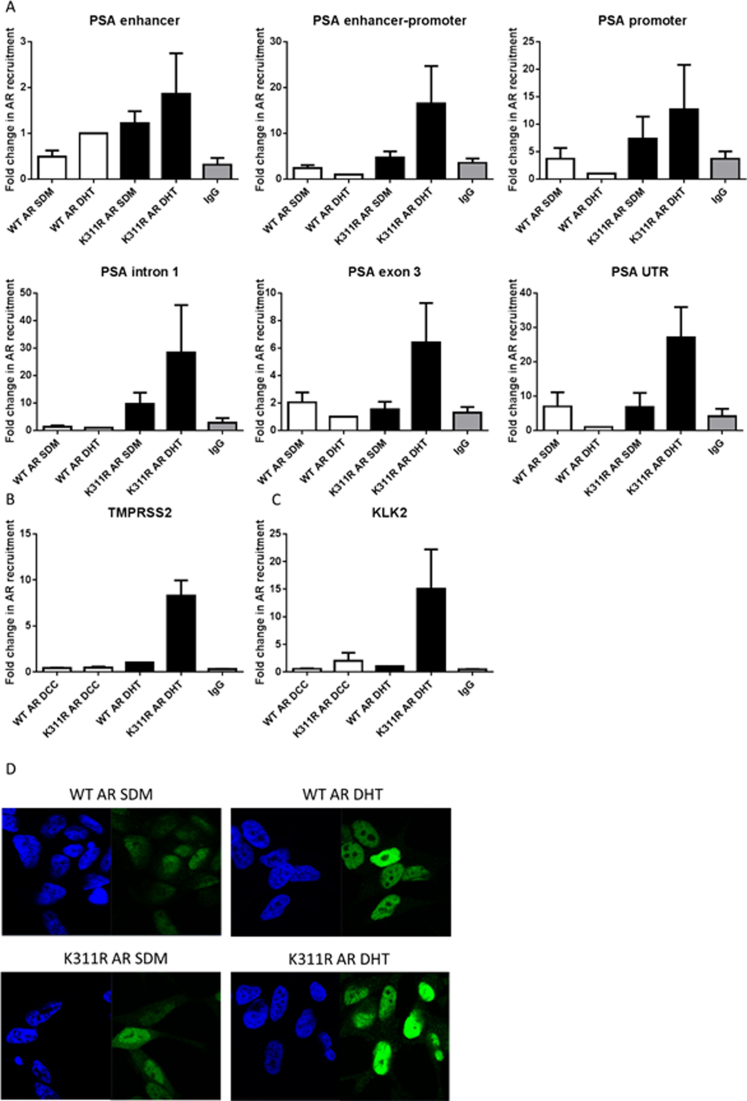
K311R AR is retained on chromatin resulting in decreased transcriptional activity. (**A**) ChIP analysis of AR recruitment to the different regions of the *PSA* gene, (**B**) promoter of *TMPRSS2* and (**C**) promoter of *KLK2* after androgen stimulation in LNCaP cells stably overexpressing WT or K311R AR after silencing endogenous AR following 120 min 10 nM DHT stimulation. Data is a mean of three independent experiments ± SEM. (**D**) LNCaP cells expressing WT or K311R AR were cultured in steroid depleted media for 70 h followed by 2 h treatment with 10 nM DHT and AR cellular localization was visualized by immunofluorescence.

To determine why K311R AR is not efficient at activating AR target gene transcription in response to androgen stimulation we performed a *PSA* ‘walking’ ChIP experiment to track its progress on chromatin. Wild type AR is recruited to the *PSA* target gene at a promoter region in response to androgen stimulation at 120 min, which is the optimal time point for AR recruitment (data not shown), and is absent in all the other regions of the gene. However, K311R AR levels at the promoter region are not changed as much in response to androgen stimulation and most importantly this mutant is retained throughout the whole *PSA* gene in response to androgen stimulation suggesting failure to effectively serve its function as a transcription factor (Figure 4A). Similar effects were observed for the *TMPRSS2* (Figure 4B) and *KLK2* (Figure 4C) genes. In agreement with the ChIP data when we analyzed the cellular distribution of AR we noted that K311R had a higher nuclear presence in the absence of androgen than the WT AR consequently the difference between the K311R AR nuclear levels in the presence and absence of AR was a lot less pronounced than observed for the WT AR (Figure 4D).

### Mutating lysine 311 of the AR causes global changes to the cellular transcriptome

**Figure 5.**
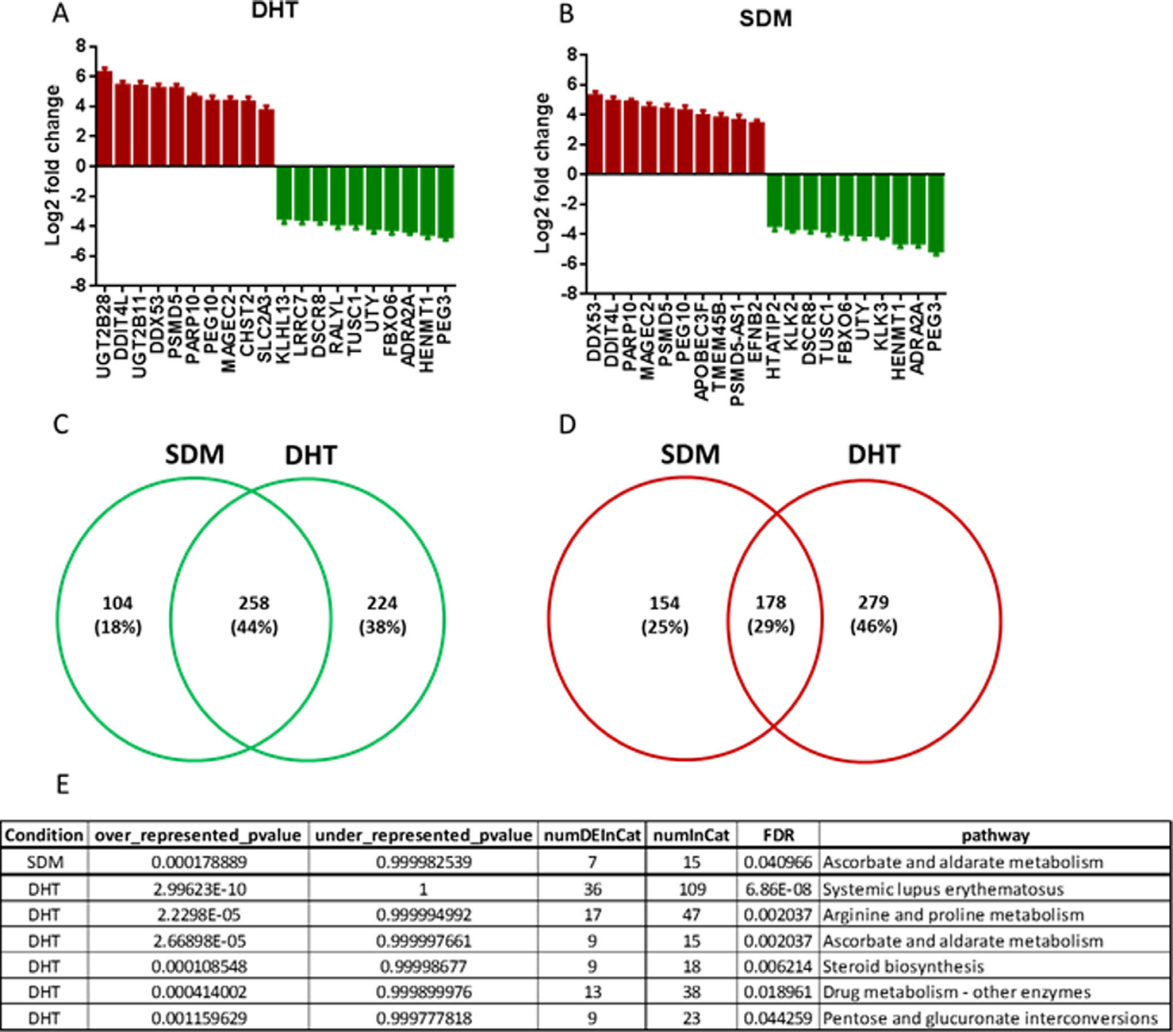
Mutation of Lysine 311 causes global changes to the LNCaP transcriptome. (**A**) RNA was sequenced in triplicate from LNCaP cells expressing WT or K311R AR and genes most significantly deregulated in the presence of DHT were plotted. (**B**) RNA was sequenced in triplicate from LNCaP cells expressing WT or K311R AR and genes most significantly deregulated in the absence of DHT were plotted. (**C-D**) Venn diagram of genes up- (**C**) and down- (**D**) regulated in K311R expressing LNCaP cells compared to WT AR expressing cells. (**E**) Table of metabolic pathways most significantly affected by mutation in K311 of the androgen receptor.

**Figure 6.**
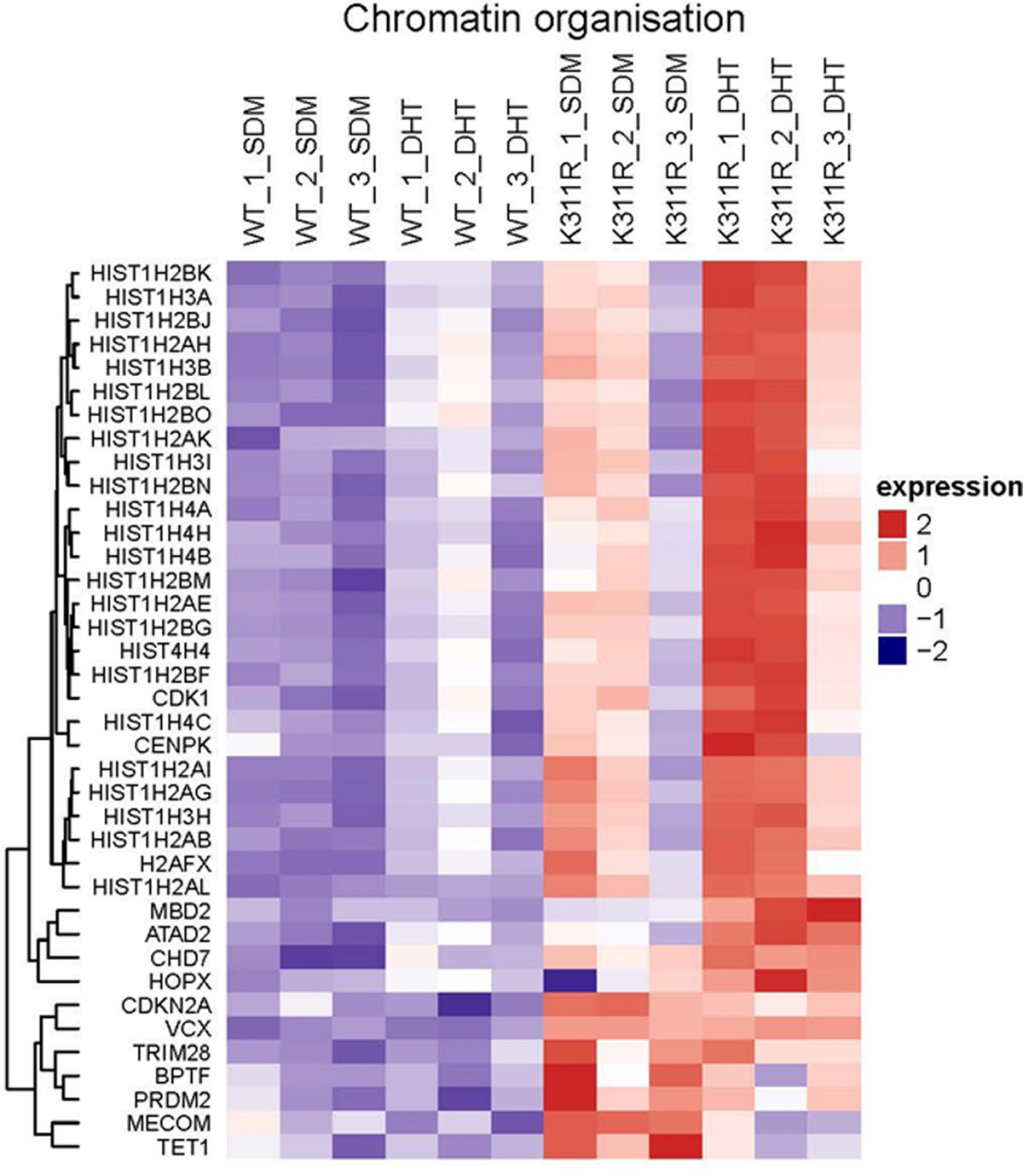
Mutation of K311 significantly upregulated the expression of genes responsible for chromatin organization. Genes significantly deregulated by K311 mutation in AR were clustered into the respective biological processes using Go term and a clustered heat map of genes involved in chromatin organization was generated.

To analyse the effects of lysine 311 mutation on PC cell transcriptome we sequenced total RNA from WT and K311R AR expressing LNCaP cells. Mutating K311 had a dramatic effect on the cellular transcriptome with 1036 genes significantly altered (adj. *p* value < 0.05) by at least 2-fold In the presence of androgen and 771 in steroid depleted conditions when compared to cells expressing WT AR. Most significantly altered genes included *KLK3* (*PSA*) and *KLK2* (Figure 5A and B). 44% of the genes downregulated by the lack of K311 were common between both conditions whilst this was the case for only 29% of significantly upregulated genes (Figure 5C and D). GO term (biological process) enrichment analysis revealed deregulation of genes in multiple biological processes (Supplementary Figure S1). Most significant changes were observed for chromatin organization with K311 mutation causing an increase in the expression of the genes from this biological process in both the absence and presence of androgen (Figure 6). This is in agreement with the increased nuclear presence (Figure 4D) and chromatin retention observed with K311R AR (Figure 4A–C). Similarly cell adhesion and motility, signal transduction and developmental pathways were all affected by mutating K311 highlighting the crucial role of this ubiquitination site for AR activity (Supplementary Figures S2–S5).

Additionally, pathway enrichment analysis revealed that abrogation of K311 ubiquitination site significantly affects cellular metabolism with ascorbate and aldarate metabolism significantly altered in both the presence and absence of androgens. Furthermore, arginine and proline metabolism (Supplementary Figure S6), steroid biosynthesis (Supplementary Figure S7) and pentose and glucoronate interconversions were affected in the presence of androgen (Figure 5E). Mutating K311 also caused a significant increase in the drug metabolism pathway. Androgen receptor activity has previously been linked to arginine metabolism with AR interacting with protein arginine methyltransferase 10 (PRMT10) (31), similarly PRTM6 was reported to co-activate the AR (32), conversely AR deficiency alters the arginine-vasopressin sexually dimorphic system (33).

### C-terminal domain of AR is required for efficient ubiquitination in cells

**Figure 7.**
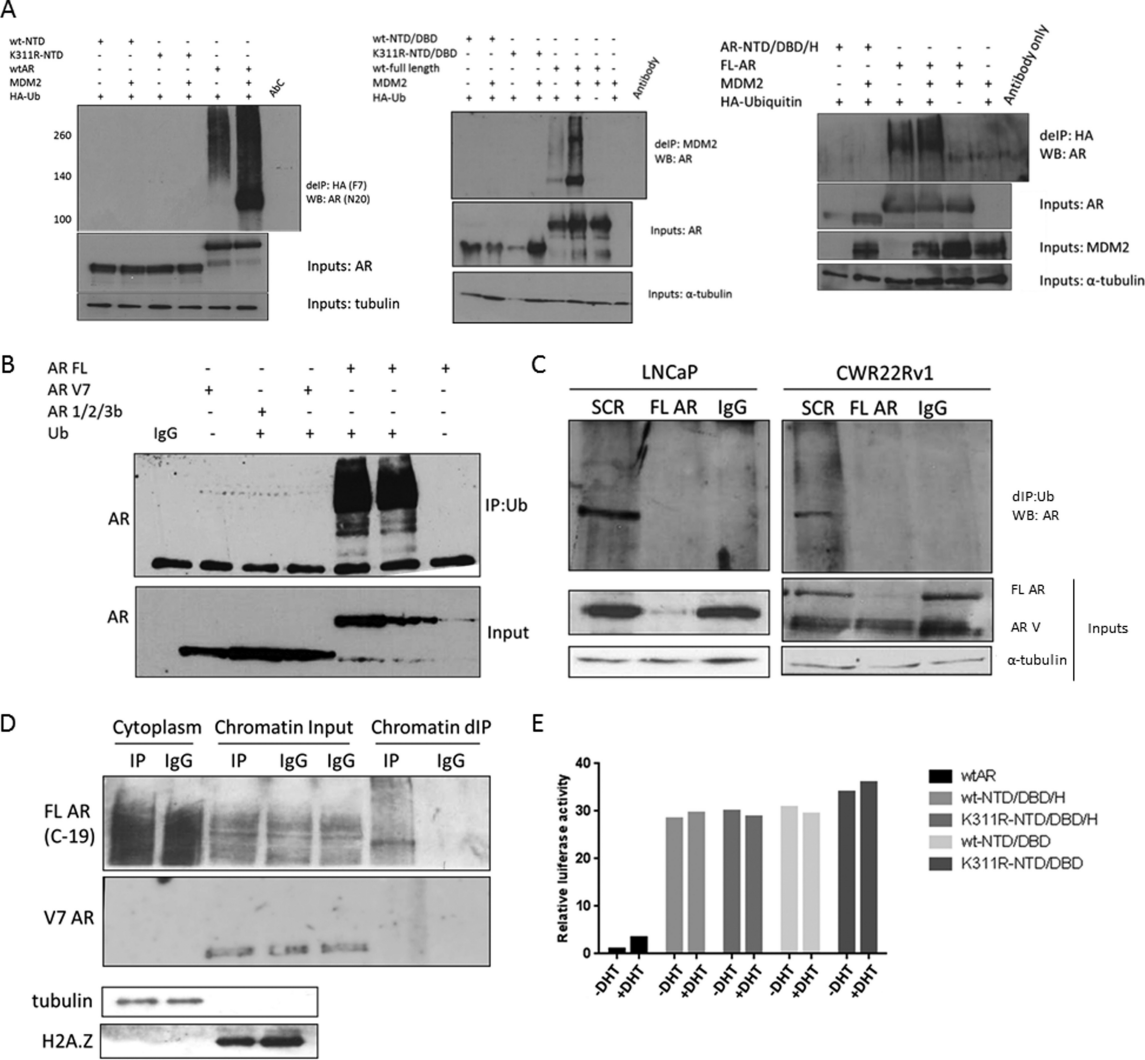
Truncated AR variants are not ubiquitinated. (**A-B**) Cells were transfected with plasmids as indicated followed by denaturing immunoprecipitation and western blotting. (**C**) LNCaP and CWR22Rv1 cells were treated with siRNAs as indicated for 96 h followed by denaturing immunoprecipitation and immunoblotting. (**D**) Chromatin and cytoplasm fractions were isolated from the CWR22Rv1 cells and denaturing immunoprecipitation was performed on each cellular fraction followed by immunoblotting. (**E**) HEK293T cells were transfected with WT AR or AR K311R along with a luciferase reporter plasmid under the control of ARE3 of the *PSA* promoter. Cells were cultured in steroid depleted media for 48 h before 24 h treatment ± 10 nM DHT. Data represent a mean of three independent experiments ± SEM. Luciferase activity was normalized to WT AR + DHT.

To assess the importance of K311 for total cellular AR ubiquitination we first overexpressed the N-terminal domain (NTD) of AR, however we failed to detect any ubiquitination of this domain by MDM2, in contrast to efficient ubiquitination of full length AR (Figure 7A, left panel). We repeated this experiment with extended AR NTD constructs that also included the DNA binding domain (DBD), NTD/DBD (Figure 7A, centre panel) and hinge (H) domain, NTD/DBD/H (Figure 7A, right panel), however we consistently were unable to demonstrate any ubiquitination of these variants in the cells even though we have previously shown that they can be successfully ubiquitinated *in vitro* (Figure 1). This observation is potentially of high clinical importance as in advanced, CRPC stage of disease it is common to see the emergence of AR truncated variants expression that lack the C-terminal ligand binding domain (34). Consequently, these variants do not require androgen to translocate to the nucleus as they lack the LBD but are constitutively active. To determine if our observation with AR domain deletion variants was clinically relevant we repeated this experiment using the AR-V7 and AR/1/2/3b variants reported in the clinic. In agreement with our previous experiment we were not able to ubiquitinate these variants within cells (Figure 7B). To confirm whether this is also true when variants are endogenously expressed we compared two prostate cancer cell lines, LNCaP cells representing androgen sensitive early disease that express only the full length AR and CWR22Rv1 cells expressing both full length AR and AR-Vs. We compared the levels of total AR ubiquitination and also ubiquitination after silencing full length AR only. In LNCaP cells, silencing full length AR abrogated AR ubiquitination as predicted because these cells only contain full length AR protein (Figure 7C). Interestingly, in CWR22Rv1 cells when we silenced only the full length AR we were not able to detect any ubiquitination of the AR-V even though we used an N-terminal AR antibody that will detect all AR-Vs. To ensure that this was not due to the fact that our denaturing immunoprecipitation protocol was biased toward proteins in the cytoplasmic compartment where unliganded full length AR may be present when AR-Vs reside on chromatin, we repeated the CWR22Rv1 experiments but we first performed cytoplasmic and chromatin extractions and denaturing IP was performed from each fraction. Using a C-terminal antibody that targets only the full length AR we could detect ubiquitinated full length AR in both the cytoplasmic and chromatin fractions, however even though AR-V7 protein was present on chromatin no ubiquitination of the AR-V7 variant was observed (Figure 7D). This indicated that even though K311 is an AR ubiquitination site, the LBD is essential for either the correct folding that allows substrate recognition by the E2/E3 or that the recognition site for E3 ligase binding AR is within the LBD, as ubiquitination frequently occurs in a region that is distinct from the substrate recognition site. To confirm our observation we compared the transcriptional activity of FL AR and truncated N-terminal ARs both with and without the functional K311 site. We observed that all of the N-terminal AR variant constructs were constitutively active and their activity was not affected by androgen stimulation, similarly their activity, unlike that of the full length AR, was not altered by lack of functional K311 (Figure 7E).

## DISCUSSION

As a key driver of PC the AR is the main therapeutic target in both early and advanced disease. However, treatments targeting the AR invariably fail and result in the development of CRPC. There are multiple mechanisms through which PC can acquire the CRPC phenotype, these include AR mutations, AR amplification and generation of AR splice variants. In addition, intracrine androgen synthesis can occur in prostate tumours and aberrant activation of alternative signaling pathways can also activate the AR. Importantly, the AR is still expressed and it continues to be a driver in CRPC and therefore it remains a viable therapeutic target (35). As such, understanding mechanisms through which AR protein activity and stability is controlled could help us identify novel therapeutic targets.

It has been previously established that AR activity and protein stability is regulated by the ubiquitin proteasome system. Previously two AR ubiquitination sites in the AF2 region of the LBD have been reported at K845 and K847. Both sites are targeted by RNF6 with K845 ubiquitination required for K847 ubiquitination and for ARA54 recruitment (11). Ubiquitination occurring at either of these sites is linked to AR transcriptional activity but is not associated with AR protein turnover. MDM2 was the first E3 identified to target AR, ubiquitination of AR by MDM2 being dependent on AR phosphorylation at S515 by CDK7 kinase via TFIIH. Additionally, MDM2 needs to be phosphorylated at S166 by AKT highlighting the complexity of the E3-substrate interaction. If AR S515 is not phosphorylated then the AR is alternatively targeted for proteasomal degradation by CHIP.

Herein we report the identification of a novel AR ubiquitination site in the N-terminal domain at position 311. The ubiquitination of lysine 311 affects AR protein stability however, its critical role appears to be in regulating AR transcriptional activity and chromatin retention. When K311 is mutated to arginine, the stability of the AR protein is increased. Similarly, mutated AR that cannot be ubiquitinated at the K311 site has dramatically reduced transcriptional activity toward androgen responsive AR target genes with global LNCaP transcriptome significantly altered and cellular metabolism pathways, including drug metabolism affected, highlighting the important role that lysine 311 plays in AR signaling. We further observe enhanced levels of K311R AR on chromatin throughout the whole androgen responsive PSA gene. We suggest this may be a consequence of defective AR cycling on and off the chromatin in response to androgen stimulation possibly because of the inability of K311R AR to bind to p300 in the presence of androgen highlighted by the increase in the expression of genes responsible for chromatin organization upon K311 mutation. We also report that AR variants that lack the LBD, including the clinically relevant AR-V7, cannot be ubiquitinated within the cell demonstrating the crucial contribution of AR LBD for E2/E3 complex binding and substrate recognition. It has been previously reported that E3 ligase binding can be affected by the presence of additional factors, Siah2 can ubiquitinate AR only when it is bound to NCOR-1 and transcriptionally inactive, this ubiquitination consequently targets AR for degradation (36). Additionally, in some cases AR needs a mediator protein to be recruited to the E3s. For AR ubiquitination by NEDD4, PMEPA1 recruits AR resulting in its ubiquitination and subsequent degradation (37). It is possible that there is a mediator protein that allows for E3 recruitment to AR that is bound in the LBD and facilitates the N-terminal ubiquitination at K311.

Mutation at K311 has not been reported in RNA-seq of PC patients (COSMIC). However, in the PC346 model derived from a primary human prostate tumour subcutaneously implanted into athymic mice that were subsequently castrated, an androgen-independent subline, PC346DCC emerged that contained the K311R AR mutation (38). Interestingly, PC346DCC cell line exhibits rapid proliferation in steroid deprived media and is unresponsive to either R1881 or OH-flutamide (39).

AR phosphorylation of serine 308 (S308), adjacent to K311, is associated with a longer time to disease specific death (40) and may impact ubiquitination at the K311 residue. Our preliminary experiments however in mutating S308 to alanine failed to impact on AR transcriptional activity in luciferase reporter assays (data not shown).

Potentially, the role of the LBD in K311 AR ubiquitination could be important if ubiquitination occurred mainly outside of the nucleus as the LBD contains a nuclear export signal (NES) which has been previously reported to signal for poly-ubiquitination and degradation but it is blocked by binding to androgens which inhibits NES (32). Consequently, in the presence of androgens, AR half-life was reported to increase in LNCaP cells from 3h to over 10h as a result of NES inhibition. However, there must be an additional role for the LBD in AR recruitment to E3s as it has been demonstrated that MDM2 can ubiquitinate AR at target gene promoters (10). The role of MDM2-mediated AR K311 ubiquitination occurring on chromatin may therefore function to promote AR chromatin dissociation and the subsequent recycling of AR to induce new rounds of androgen responsive gene transcriptional activity.

## Supplementary Material

Supplementary Data
