# Supplementary material for: Identification of a novel K311 ubiquitination site critical for androgen receptor transcriptional activity
Source: Nucleic Acids Res. 2016 Nov 28;45(4):1793–804. doi: 10.1093/nar/gkw1162 (PMC5389688; doi:10.1093/nar/gkw1162)

Suppl Figure 1. Clustering of genes into the biological processes significantly altered by the K311 mutation of AR

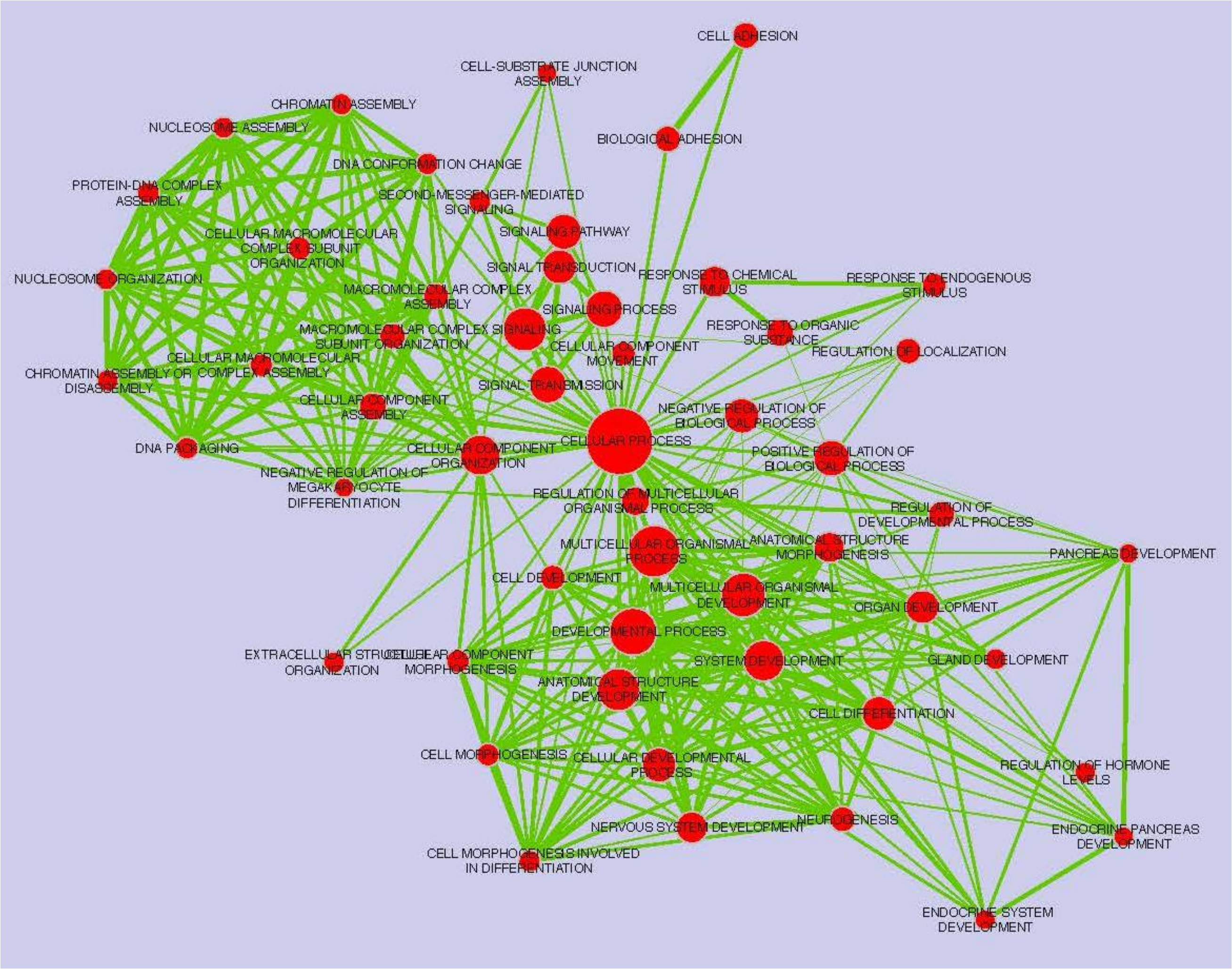

Suppl Figure 2. Clustered heat map of genes responsible for cell adhesion affected by K311 mutation

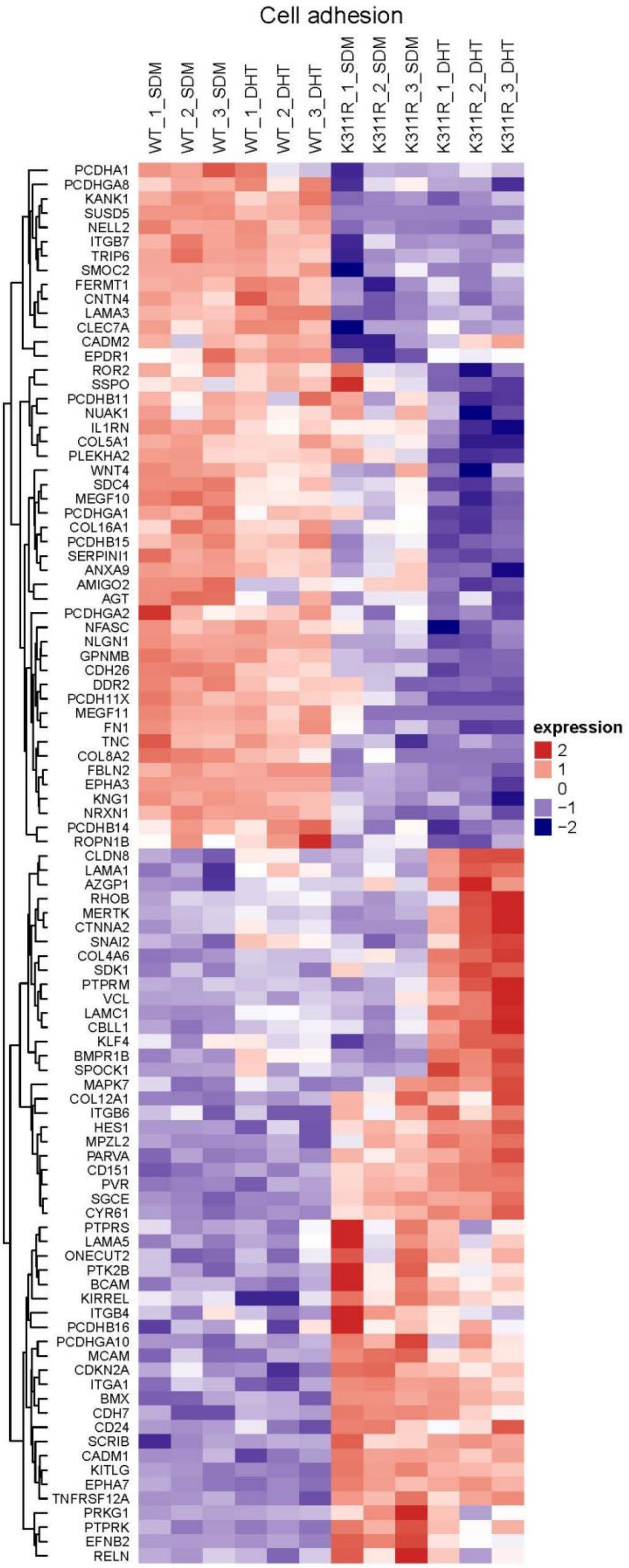

Suppl Figure 3. Clustered heat map of genes responsible for cell motility affected by K311 mutation

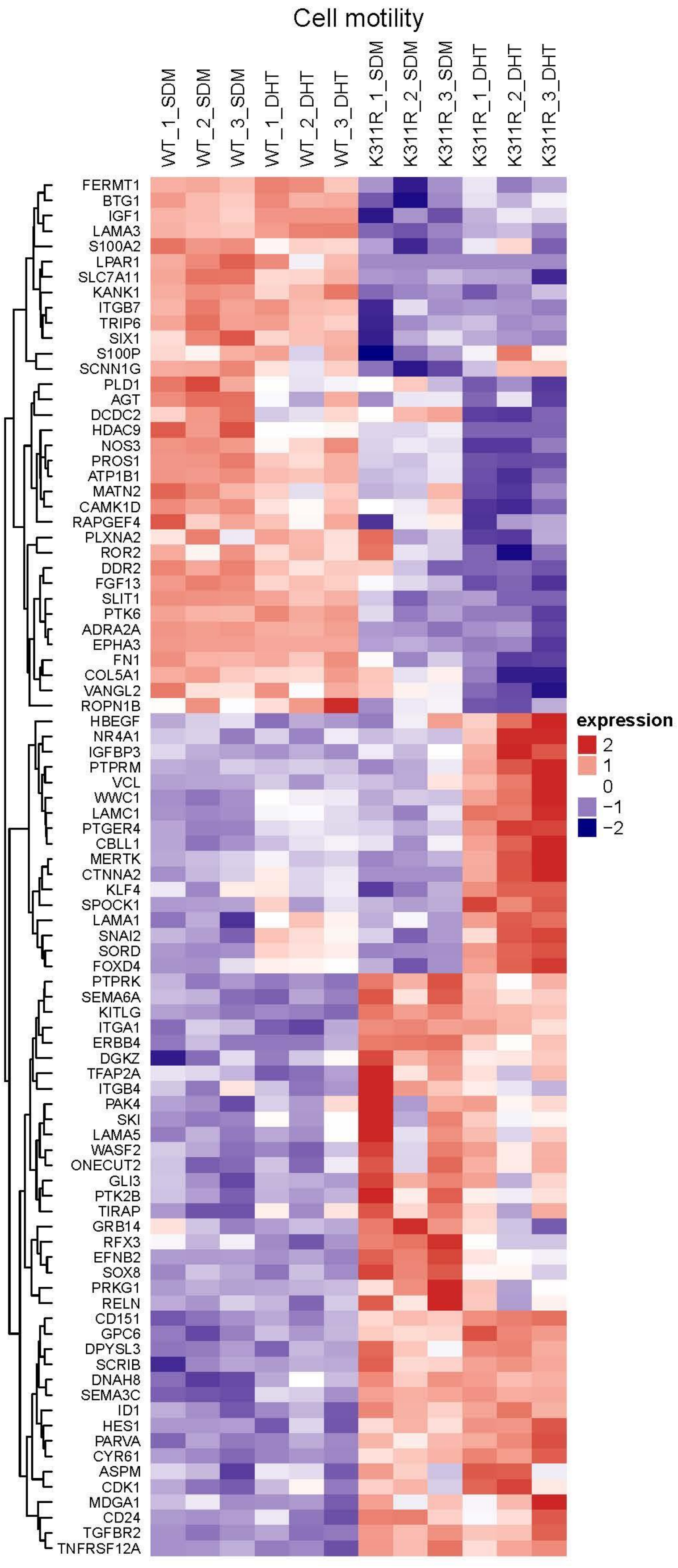

Suppl Figure 4. Clustered heat map of genes responsible for signal transduction affected by K311 mutation

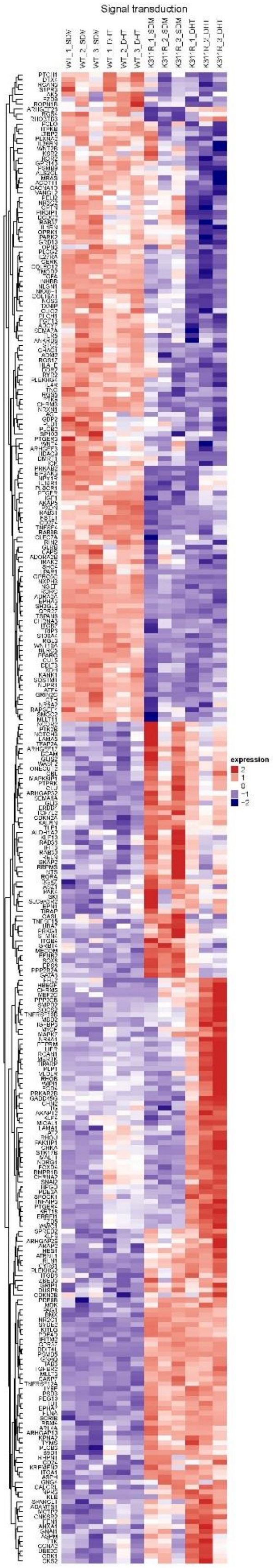

Suppl Figure 5. Clustered heat map of genes responsible for development affected by K311 mutation

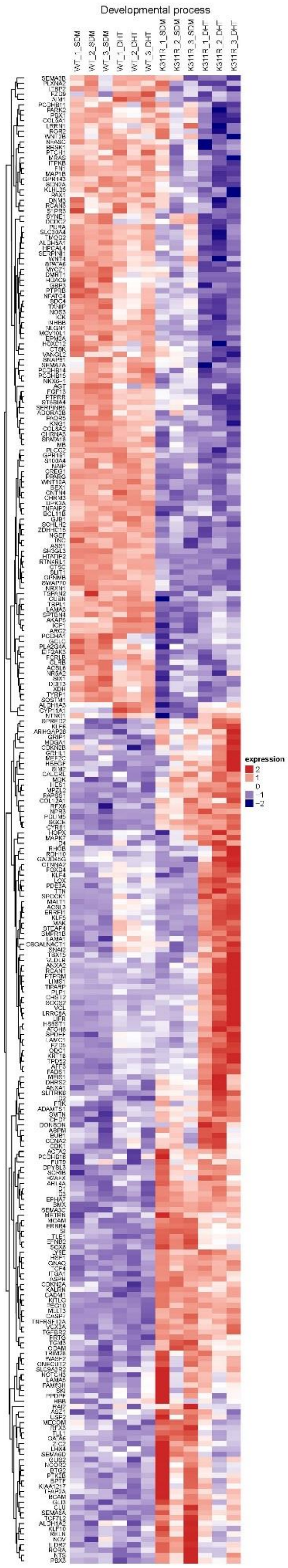

Suppl Figure 6. The effect of K311 mutation on arginine and proline metabolism in the presence of androgen

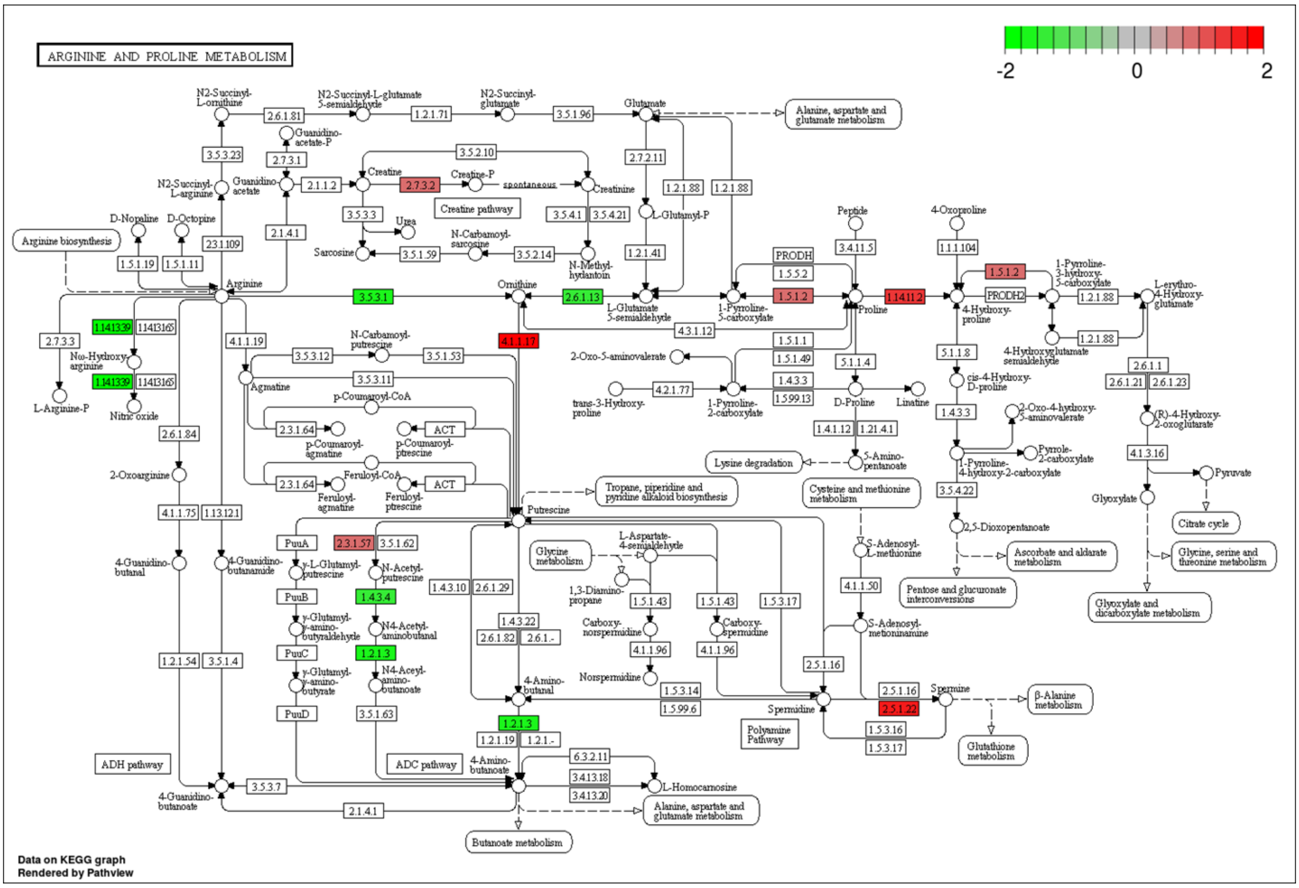

Suppl Figure 7. The effect of K311 mutation on steroid biosynthesis in the presence of androgen.

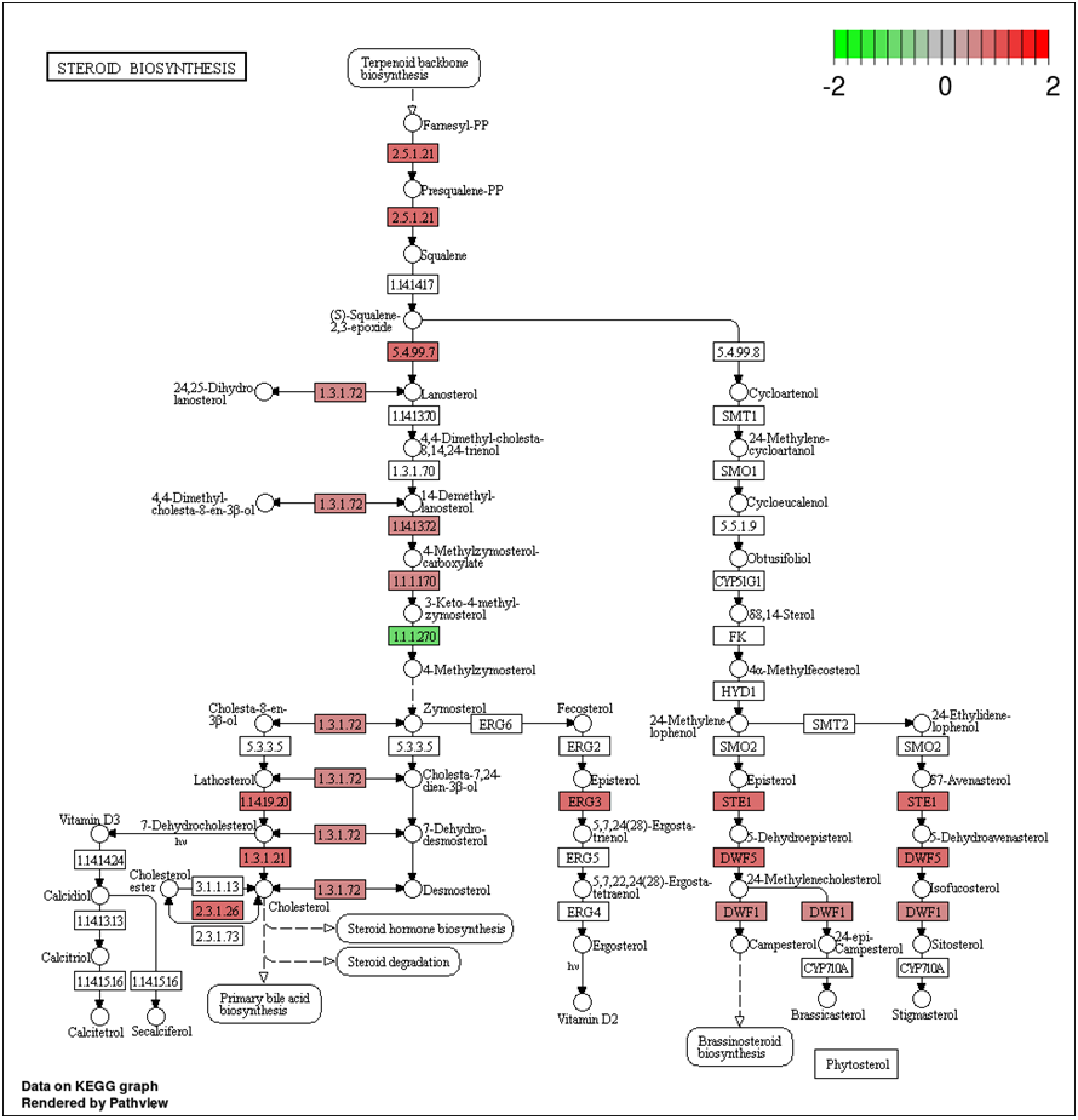

Supplement: Supplementary Data [file gkw1162_Supp.pdf]
